# Wastewater surveillance to track influenza viruses

**DOI:** 10.2471/BLT.24.292285

**Published:** 2024-09-01

**Authors:** Leshan Xiu, Kun Yin

**Affiliations:** a School of Global Health, Chinese Center for Tropical Diseases Research, Shanghai Jiao Tong University School of Medicine, 227 South Chongqing Road, Shanghai 200025, China.

The threat posed by the highly pathogenic avian influenza virus strain H5N1 to humans is growing. Initially detected in China, migratory birds have carried and disseminated H5N1 across multiple continents, causing destructive poultry outbreaks globally.^1^ In affected areas, H5N1 has spilled over into 41 aquatic and terrestrial mammalian species, including wild and domestic animals. Although human infections with H5N1 remain relatively rare, with 889 cases reported across 23 countries from 2003 to 2024, the high case fatality rate of 52.1% (463/889) is alarming.^2^ The potential for viral evolution and human exposure leading to sustained human-to-human transmission poses a public health threat.^3^ Therefore, implementing a comprehensive dashboard for tracking H5N1 is crucial for understanding the prevalence of the disease in various populations and regions.

Adopting a One Health approach that integrates human, animal and environmental surveillance, can enhance our ability to monitor H5N1 transmission. This integrated surveillance provides an early warning system and informs targeted interventions. However, the global burden of H5N1 in animal and human populations remains unclear due to insufficient active surveillance, and the transmission dynamics and risks associated with different animal groups are not well understood.^4^ Large-scale surveillance of both human and animal populations remains challenging for many countries. Alternative tools for the rapid identification, containment and reduction of H5N1 spread are critical. Environmental surveillance, which involves the systematic collection of samples and data on pathogens from the environment to inform public health decisions, has a long-standing tradition.^5^^–^^7^ Wastewater surveillance has been effectively used to monitor the population-level spread of infectious diseases such as coronavirus disease 2019 (COVID-19), mpox and polio, showing strong correlations between pathogens detected in wastewater and clinical disease burden.^5^^,^^7^ Although it provides a broad indication of influenza spread, wastewater surveillance can guide public health decision-making by identifying hotspots and assessing the outbreak’s trend. However, numerous factors and bottlenecks could affect the accuracy of such information. 

Although academic wastewater surveillance programmes for infectious diseases have shown early success, most countries are poorly equipped to conduct wastewater surveillance on a nationwide scale.^5^ Implementing environmental surveillance in lower-resource settings, where a significant portion of the population resides in homes not connected to formal wastewater treatment plants, has been hampered by epidemiological and resource challenges.^6^ For instance, a recent study estimates that less than 40% of wastewater is treated in India, and in some low-income countries, treatment rates may be below 10%.^5^ Even in high-income countries like the United States of America, only 80% of the population is served by a piped sewage network.^7^ Additionally, grey and black water from poultry and livestock farms may be discharged into nearby water systems without treatment. While the current risk of infection for the public remains low, as the H5N1 virus spreads, wastewater surveillance could help identify where testing efforts should be intensified in cattle, other animals and potentially humans. Expanding the sources of wastewater monitored and addressing these disparities is crucial to ensuring equitable access to actionable surveillance data for all communities, especially in resource-limited settings.

The major bottleneck in wastewater surveillance is identifying the source of H5N1. Interpretation of wastewater data is further hindered by the inability to distinguish H5N1 from other influenza A subtypes. The National Wastewater Surveillance System in the United States aims to detect traces of circulating infectious diseases by collecting and analysing wastewater data from communities across the country. While current wastewater surveillance methods under this national system can detect influenza A viruses, they struggle to differentiate H5N1 from other influenza A subtypes. Moreover, wastewater testing cannot pinpoint the exact source of the influenza A virus,^8^ which may originate from humans, animals or animal products. Following the detection of the virus in cow workers, WastewaterSCAN, a Stanford and Emory University initiative, launched an H5 avian influenza wastewater dashboard. This system detected fragments of H5N1 genetic material in the city’s wastewater. However, the system was unable to definitively identify the species responsible for carrying the H5N1 virus.^9^ Enhancing existing monitoring methods, as well as conducting virus isolation and culture, holds the potential to accurately identify H5N1 subtypes. By conducting genomic comparisons and identifying gene variations associated with host adaptation, the origin of the virus could be determined.^10^

Policy-makers and governments should prioritize the expansion of wastewater surveillance systems to include a broader range of environmental and animal sources. Strengthening international collaboration and data sharing to improve our understanding of H5N1 transmission dynamics and ensuring timely and coordinated responses to outbreaks are also essential. 
